# An Approximate Bayesian Estimator Suggests Strong, Recurrent Selective Sweeps in Drosophila

**DOI:** 10.1371/journal.pgen.1000198

**Published:** 2008-09-19

**Authors:** Jeffrey D. Jensen, Kevin R. Thornton, Peter Andolfatto

**Affiliations:** 1 Section of Ecology, Behavior and Evolution, University of California San Diego, La Jolla, California, United States of America; 2 Department of Ecology and Evolutionary Biology, University of California Irvine, Irvine, California, United States of America; 3 Department of Ecology and Evolutionary Biology, Princeton University, Princeton, New Jersey, United States of America; 4 Lewis-Sigler Institute for Integrative Genomics, Princeton University, Princeton, New Jersey, United States of America; University of Oxford, United Kingdom

## Abstract

The recurrent fixation of newly arising, beneficial mutations in a species reduces levels of linked neutral variability. Models positing frequent weakly beneficial substitutions or, alternatively, rare, strongly selected substitutions predict similar average effects on linked neutral variability, if the product of the rate and strength of selection is held constant. We propose an approximate Bayesian (ABC) polymorphism-based estimator that can be used to distinguish between these models, and apply it to multi-locus data from *Drosophila melanogaster*. We investigate the extent to which inference about the strength of selection is sensitive to assumptions about the underlying distributions of the rates of substitution and recombination, the strength of selection, heterogeneity in mutation rate, as well as the population's demographic history. We show that assuming fixed values of selection parameters in estimation leads to overestimates of the strength of selection and underestimates of the rate. We estimate parameters for an African population of *D. melanogaster* (*ŝ*∼2E−03, 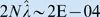) and compare these to previous estimates. Finally, we show that surveying larger genomic regions is expected to lend much more discriminatory power to the approach. It will thus be of great interest to apply this method to emerging whole-genome polymorphism data sets in many taxa.

## Author Summary

Understanding the process of adaptive evolution requires quantifying the extent to which beneficial mutations contribute to differences between species. However, fundamental parameters of adaptation, such as the rate and strength of beneficial mutations, are poorly understood and have historically been difficult to estimate from data. In particular, distinguishing a high rate of weakly selected substitutions from a low rate of strongly selected substitutions has been problematic. Here, we introduce a new method to estimate the parameters of adaptive evolution from multi-locus population genetic data. We conduct simulations to show that this method is able to discriminate the rare/strong model from the frequent/weak model. Applying this method to an African population sample of *Drosophila melanogaster*, we estimate selection parameters and find that recurrent adaptive evolution has reduced genome variability by ∼50% on average. The availability of genome-scale population genetic data will lend considerable discriminatory power to the approach. Thus, this new approach represents an important step towards characterizing the nature of adaptive evolution in natural populations.

## Introduction

The fixation of beneficial mutations can strongly reduce levels of closely linked neutral variation – the so-called genetic hitchhiking effect [1]. This prediction has been used to search for positive selection by looking for regions of the genome with reduced variability [*e.g.*, 2]. The hitchhiking model most often used is of a single selective sweep, where the location and timing of selection are assumed to be known [3]. This single sweep model has been of great value in understanding the effect that a single selective event has on patterns of polymorphism, as a function of the strength of selection and location of the beneficial mutation [*e.g*., 1],[4],[5]. However, this model is somewhat disconnected from the problem of detecting selective sweeps in the genome, for which locations and timings are not known *a priori*, and should be treated as random variables.

Kaplan *et al.* (1989) described a “recurrent hitch-hiking” (RHH) model, where the expected number of sweeps (per base pair, per 2*N* generations) is *2Nλ* with sweeps occurring at random locations in the genome [6]. The RHH model is most commonly considered for the case of genic selection on new mutations entering the population [*e.g.*, 6]–[8]. Under this model, several patterns expected under the single sweep model no longer apply. For example, the single sweep model predicts coalescent histories with long internal branches, as some lineages may escape the recent coalescent event via recombination. This results in the widely employed prediction of an excess of high-frequency derived alleles flanking the fixed site [5]. Under RHH models however, the probability of such a history is small, as sweeps are on average old and high frequency derived mutations have thus likely drifted to fixation [9].

Wiehe and Stephan (1993) showed that under a RHH model, for a given recombination rate, the expected level of heterozygosity at linked sites relative to neutral expectations is dependent upon the compound parameter (*s*)(2*Nλ*), where 2*Nλ* is the rate of fixation of beneficial mutations and *s* is the average strength of selection [7]. This result implies that that the two parameters are confounded (much like the effective population size, *N_e_*, and mutation rate, *μ*, in *θ* = 4*N_e_μ*) as their effect on expected levels of diversity depends on their product. In *D. melanogaster* and *D. simulans*, lower than expected levels of nucleotide diversity are observed in regions of reduced recombination [10] and in the coding sequences of rapidly evolving proteins [11],[12]. These findings are compatible with either strong but infrequent positive selection (*i.e.*, large *s* and small 2*Nλ*) or weak but common positive selection (*i.e.*, small *s* and large 2*Nλ*) [7], [11]–[13].

A number of methods have been proposed for quantifying *s* and 2*Nλ* (separately) using divergence and polymorphism data [*e.g.*, 11]–[12], [14]–[17]. These approaches typically make strong assumptions regarding the possible distribution of selection coefficients, the number of adaptive substitutions between species, or the timing of selection. For example, Li and Stephan (2006) examined 250 non-coding regions from an East African population of *D. melanogaster* [18]. Using a likelihood approach, they estimate that approximately 160 beneficial mutations have fixed in this population over the last ∼60,000 years (corresponding to 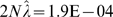), with mean selection coefficient *ŝ*∼0.002. This inference is achieved by effectively assuming that the timing of all sweeps is known (and the time since the sweep, *τ* = 0). Under a recurrent sweep model, this assumption may bias the estimation of *s* and *2Nλ*. Additionally, as this method relies on first fitting a demographic model to non-coding DNA polymorphisms, it is possible that the effects of purifying selection on the site frequency spectrum of non-coding DNA [19]–[20] may strongly affect the estimates.

Using synonymous polymorphism data in *D. melanogaster*, and divergence to *D. simulans*, at 137 X-linked loci, Andolfatto (2007) employed a maximum likelihood approach to estimate the joint parameter *2Nλs*, followed by a McDonald-Kreitman-based method to separately estimate *2Nλ* and *s* [11]. Based on these calculations, Andolfatto estimated that most beneficial amino acid substitutions are very weakly advantageous on average (with average *ŝ*∼1.2E−5 and 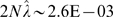). Macpherson *et al.* (2007), using polymorphism data from *D. simulans* (and divergence to *D. melanogaster*), propose a method to infer the rate and strength of selection from the spatial scale of variation in polymorphism and divergence [12]. In contrast to Andolfatto's estimates, Macpherson *et al.* estimate a much stronger average selection coefficient (*ŝ*∼0.01) and less frequent selection (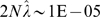). However, they note that their method is more likely to detect strong selection, so the effects of many weakly beneficial mutations may be missed.

By evaluating a wide array of recurrent selection models across a variety of sampling schemes, with parameters relevant for both Drosophila and human populations, we demonstrate here that there are differences in the predictions of weak and strong selection models, both in the spatial distribution of variability levels and the distribution of polymorphism frequencies (also called the site frequency spectrum, hereafter SFS). We propose a polymorphism-based approximate Bayesian (ABC) estimator that is most closely allied to the approach of Macpherson *et al.* (2007), but is also applicable to sub-genomic multi-locus data of the kind that has most often been collected [*e.g.*, 11], [21]–[22], and incorporates more information from the data. Fundamentally, this estimation procedure is based on the principle that while models may predict the same average affects, the variance of many common summary statistics varies greatly between models. We show that highly accurate estimation will be possible with large-scale genome polymorphism data, and that the approach is robust to both mutation and recombination rate heterogeneity.

## Results/Discussion

### Distinguishing Models of Weak and Strong Recurrent Selection

**Figure 1:**
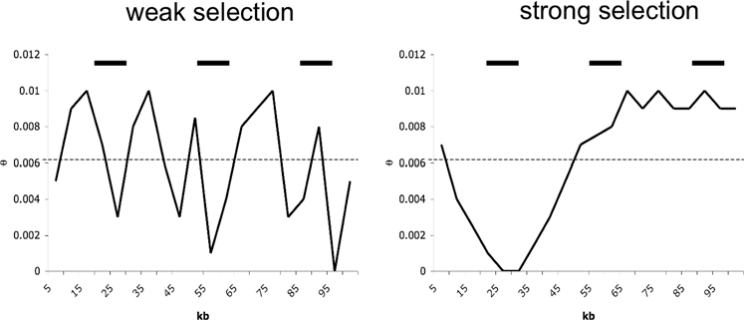
A cartoon representation of the difference between models of common weak and rare strong selection. On the X-axis is distance along a chromosome in kilobases (kb), and the on the Y-axis is variability. The dotted-line represents the average heterozygosity, and the solid bars represent loci sequenced for polymorphism data. As shown, under the weak selection model each individual selective fixation impacts a small genomic region, though sweeps are occurring frequently. The combination results in a homogenizing effect across the chromosome. Alternatively, under the strong selection model each fixation impacts a large genomic region. However, because selection is rare, other regions will appear at equilibrium. Thus, sampling loci under these models, the mean level of variation among loci may be identical, but the variance between loci will be far greater under the strong selection case – with some loci falling in severely reduced regions of variation, and others in neutral regions.

As pointed out by Macpherson *et al.* (2007), there is reason to anticipate that region size may be key in uncoupling the strength of selection (*s*) from the rate of beneficial fixation (2*Nλ*) (see Table 1 for a summary of terms). Intuitively, because only a very strong sweep is capable of severely reducing larger regions - on the order of 100 kb for instance - regions may be observed with very little variation under this model. However, because selection is rare, other regions will appear close to neutral. Conversely, weak selection serves to homogenize variation as it occurs with much greater frequency. For example, for an effective population size of 10^6^ and *ρ* = 4*Nr* = 0.1/bp, the expected waiting time between sweeps is 68,000 generations, for *s* = 1E−04 and 2*Nλ* = 5E−04, for a region size of 10^4^ base pairs. For the same population parameters, but *s* = 0.01 and 2*Nλ* = 5E−06, the expected waiting time between sweeps is 532,000 generations. Considering that most signatures of selection are dissipated by 400,000 generations for these parameters [9],[23], this demonstrates that if selection is strong and rare on average, there will likely be a large variance across the genome, from strongly swept to essentially neutral looking regions (Figure 1). Capturing this variance is dependent upon the size of the sampled region as, while many values of *s* may reduce a 500 bp region for instance, only large selection coefficients are capable of reducing a 100 kb region, suggesting that larger region sizes should afford greater discriminatory power.

**Table 1:** Definitions of commonly used symbols.

Symbol	Definition
*τ*	Time since sweep in units of 4*N* generations
*L*	The length of the sequenced region
*n*	Sample size
*θ*	4*Nμ*; the population mutation rate
*ρ*	4*Nr*; the population recombination rate
*s*	The selection coefficient of beneficial mutations
2*Nλ* = *Λ*	The rate of fixation of beneficial mutations

**Figure 2:**
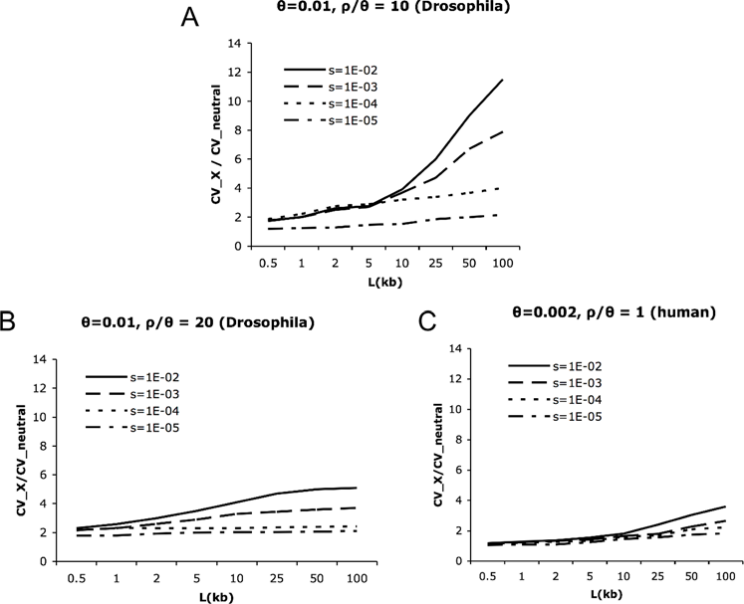
The ratio of the coefficient of variation (CV) of *π* under four recurrent selection models to the CV of *π* under equilibrium neutrality, for four selection coefficients (*s* = 1E−02, 1E−03, 1E−04, and 1E−05). *n* = 25. A) Drosophila-like parameters, *ρ*/*θ* = 10, *ρ* = 0.1/site, *θ* = 0.01/site. (B) Drosophila-like parameters, *ρ*/*θ* = 20, *ρ* = 0.2/site, *θ* = 0.01/site. (C) Human-like parameters, *ρ*/*θ* = 1, *ρ* = 0.002/site, *θ* = 0.002/site. The selection coefficient, *s*, and rate of advantageous substitution, 2*Nλ*, differ among selection models, though their product remains the same for each given value of *ρ/θ* (sλ = 2.5E−13 for *ρ/θ* = 10, 20; sλ = 5E−11 for *ρ/θ* = 1 and *N* = 10^6^). 1000 replicates were generated under each model for each data point. As seen, the models begin to differentiate from one another as the size of the sampled region gets larger, suggesting greater power to distinguish weak and strong selection models at larger physical scales.

In order to more precisely determine this ‘region size’ effect, we examined 500 bp, 1 kb, 2 kb, 5 kb, 10 kb, 25 kb, 50 kb, and 100 kb regions using simulated data (Figure 2A). First examining *L* = 500 bp regions (matching existing empirical datasets, *e.g.*, [11],[21]), we observe that there is relatively little difference in the coefficient of variation (CV) of *π* between RHH models of strong and weak selection (Figure 2), consistent with previous observations that *s* and *2Nλ* are difficult to estimate separately with data of this kind [13].

Examining larger regions, the CV is essentially unchanged under weak selection models once regions larger than 25 kb have been sequenced. Conversely, the CV continues to grow rapidly under a strong selection model, producing a four-fold difference in the CV at 50 kb of sequence relative to weak selection models, and over a five-fold difference at 100 kb for these parameters, for Drosophila-like parameters (*θ* = 0.01/site; *ρ/θ* = 10). The difference between strong and weak selection models in Figure 2 does not appear to be attributable to the total amount of surveyed sequence between the 100 kb and 500 bp regions. By comparing the distribution observed when considering ten 100 kb regions vs. two thousand 500 bp regions (and thus the same number of segregating sites on average) we still observe a large difference in CV at the scale of 100 kb, and little difference between models at the scale of 500 bp (results not shown).

We found that the relative point at which the region size benefit plateaus is a function of *θ*, *ρ*/*θ*, 2*Nλ* and *s*. We examined the effect of doubling the recombination rate (such that *ρ/θ* = 20), and find that the CV is reduced under all models relative to *ρ/θ* = 10, and that the models begin to differentiate at smaller region sizes (Figure 2B). These effects are a result of the fact that the expected size of the swept region will decrease as the recombination rate increases [6]. Additionally, using human-like parameters (*θ* = 0.002/site, *ρ/θ* = 1), we find that the pattern of an increasing CV with region size is still observed to some extent. However, the CV is much larger on average even under neutrality when *ρ/θ* = 1, and the models are more similar to one another with human-like parameters (Figure 2C) than with Drosophila-like parameters (Figures 2A and B). This implies that weak and strong selection models will be more difficult to distinguish in humans.

**Figure 3:**
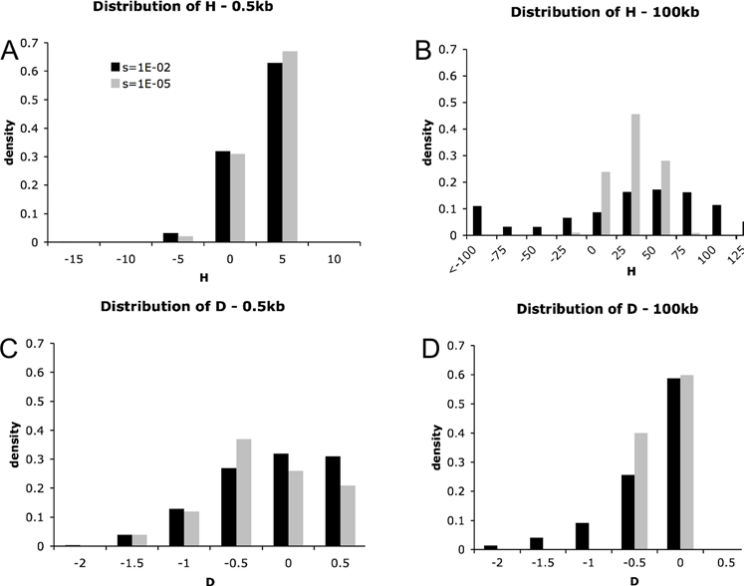
Distributions of Fay and Wu's *H*-statistic [5] and Tajima's *D*-statistic [45] under common weak and rare strong selection models. (A) The distribution of Fay and Wu's *H* for 500 bp regions. (B) The distribution of Fay and Wu's *H* for 100 kb regions. (C) The distribution of Tajima's *D* for 500 bp regions. (D) The distribution of Tajima's *D* for 100 kb regions. 1000 replicates were generated under each model and the following parameters were fixed: *ρ* = 0.1/site, *θ* = 0.01/site (thus, *ρ*/*θ* = 10), and *n* = 25. The selection coefficient, *s*, and rate, 2*Nλ*, differ among models, though their product is the same (*2Nλs* = 5.0E−07). As shown in [9], the mean *H* is positive under a recurrent sweep model. However, while we confirm that the means are positive and nearly identical for 2*Nλs* = constant, we find that previous attempts to differentiate these models have likely been hampered by the scale of the regions considered. Specifically, while the distributions for both statistics appear similar for 500 bp regions, they are quite distinct at larger physical scales (*i.e.*, 100 kb).

It is noteworthy that for large surveyed regions, more strongly negative values of Fay and Wu's *H*-statistic (*i.e.*, SFS skewed towards high-frequency derived alleles) and Tajima's *D*-statistic (*i.e.*, SFS skewed towards rare alleles) are observed under strong selection models (Figure 3), suggesting that differences in the polymorphism site frequency spectrum may also be used to distinguish between models if large enough regions are surveyed. Though this differs qualitatively from the conclusions of Przeworski (2002), simulations demonstrate that this is attributable to a modeling difference (results not shown), as we here allow sweeps within the sampled region (following [24]). This discrepancy between modeling approaches will thus only become greater as region sizes increase.

### Estimating Recurrent Selection Parameters: An Approximate Bayesian Approach

The above results suggest that focusing on variability across loci may distinguish models of strong, rare sweeps from those of frequent, weak sweeps. Thus, we here implement an approximate Bayesian (ABC) approach to estimate the strength of selection (*s*), the rate of fixation of beneficial mutations (2*Nλ*) and the neutral population mutation rate (*θ = 4N_e_u*) under a recurrent hitchhiking model. We begin by employing the observed mean and standard deviation of heterozygosity (*π*), which is closely related to previously published estimation procedures [*e.g.*, 11]–[12]. In order to evaluate this approach, we tested the performance using simulated data. Figure S1 shows distributions of maximum *a posteriori* (MAP) estimates of *s*, 2*Nλ*, and *θ* under two different models (strong rare and weak frequent selection), for 50 kb and 500 bp regions. In these simulations, *s*, 2*Nλ* and *ρ* have fixed values indicated with the vertical dotted line.

We find that this *π*-based estimation performs reasonably well, particularly when the size of surveyed regions is large and selection is strong. For 500 bp regions, MAP estimates are accurate within an order of magnitude. However, distributions of MAP estimates are typically widely dispersed, particularly when selection is weak (Figure S1; Table S1). Additionally, estimation of *s*, 2*Nλ*, and *θ* is generally upwardly biased. Under the best conditions - large region sizes and strong selection - the performance of the estimator is greatly improved (RMSE(*ŝ*) = 0.179, and the relative bias, RB(*ŝ*) = −0.281).

Given the computational efficiency of ABC, it is straightforward to explore multiple combinations of test statistics, in order to determine whether incorporating additional information from the site frequency spectrum or spatial distribution of sites may significantly improve the accuracy of estimation. We found that the incorporation of the mean and variance of several common summary statistics did not significantly improve or alter estimation, owing to correlations with *π* (results not shown). However, other statistics such as *θ_H_* [25], and *ZnS* [26] are only weakly correlated with *π* (results not shown). As such, it may be anticipated that the addition of these statistics may provide additional information, which would allow for further discrimination between models.

**Figure 4:**
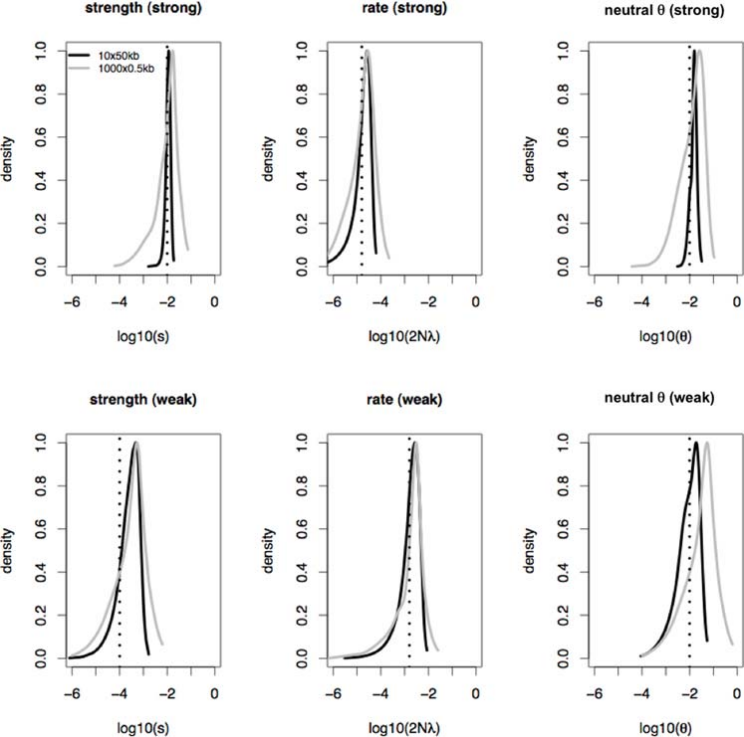
Approximate Bayesian estimation of the strength and rate of selection as well as the neutral *θ*, when estimation is based upon the means and SDs of *π*, *S*, *θ_H_* and *ZnS*. The model is one in which *s* and 2*Nλ* are fixed. For the strong selection case *s* = 1.0E−02, and 2*Nλ* = 2.0E−05, for weak selection *s* = 1.0E−04, and 2*Nλ* = 2.0E−03. *ρ* = 0.1/site and *θ* = 0.01/site. Shown are the distributions of 1000 MAP estimates. The dotted lines indicate the true values. The distributions for 10 50 kb region datasets are given in black, and for 1000 500 bp datasets in gray. As shown, the use of these multiple summary statistics improves estimation relative to *π* alone (Figure S1), reducing the RMSEs (Table S1).

This intuition appears to be accurate. The addition of the mean and SD of *ZnS* and *θ_H_* particularly, and the number of segregating sites (*S*) to a lesser extent, appear to improve the performance of the method considerably. For strong selection, even at the 500 bp scale, the addition of multiple summary statistics reduces the bias and RMSE by half relative to *π*-based estimation (Table S1), thereby improving the accuracy of estimation (Figure 4). This result suggests a distinct advantage to utilizing these additional summary statistics, particularly when surveying larger regions.

### The Effect of Variation in Model Parameters

Though the parameters *s*, 2*Nλ*, and *ρ* are fixed in the above simulations, these parameters likely vary among genomic regions in real data. While it is attractive to assume a fixed parameter model given its simplicity, if the true model is in fact one in which parameters are drawn from distributions, this may lead to a bias in estimation owing to misspecification of the model. We consider a variety of examples – those in which *s* and 2*Nλ* are drawn from exponentials, and *ρ* is drawn from an exponential or normal. When comparing between fixed and distributed models – the mean of the distribution is equal to the fixed value used previously (*i.e.*, if in the fixed model *s* = 0.01, the distribution model to which it would be compared would have *s* exponentially distributed with mean 0.01). Figure S2 documents the effect of modeling parameters drawn from distributions on the relative CV of π (compare to Figure 2). As expected the relative CV is inflated compared to the fixed parameter model, which may lead to biases in estimation if unaccounted for.

**Figure 5:**
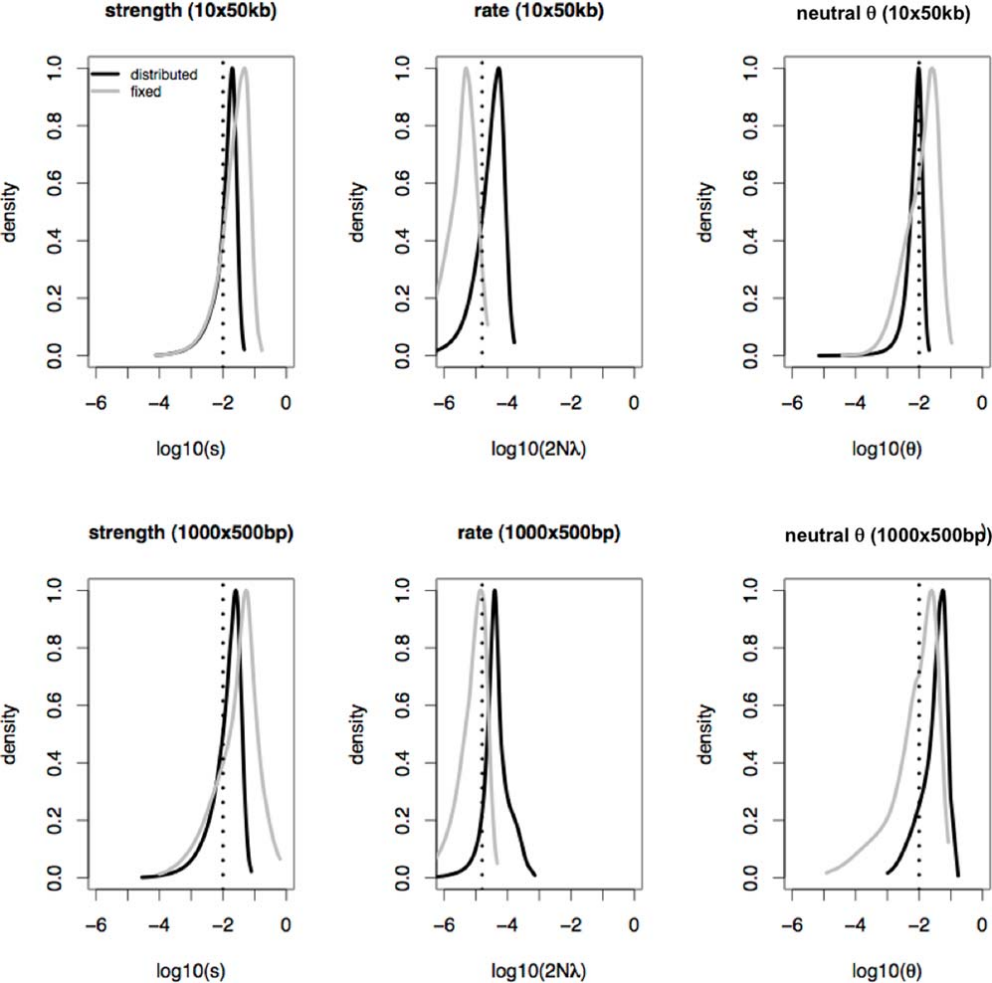
Approximate Bayesian estimation of the strength and rate of selection as well as the neutral *θ*, when estimation is based upon the means and SDs of *π*, *S*, *θ_H_* and *ZnS*. The true model is one in which *s* and 2*Nλ* for each locus is drawn from exponential distributions. The mean *s* = 1.0E−02, and the mean 2*Nλ* = 2.0E−05 (given by dotted lines). Shown are the distributions of 1000 MAP estimates. *ρ* is given by a Normal(0.1, 0.05), and *θ* is fixed at 0.01/site. Results are given for estimation when priors are constructed under a distributed parameter model, as well as a fixed parameter model (see Methods), for 10×50 kb and 1000×500 bp regions. As shown, falsely assuming fixed selection parameters leads to consistent biases in estimation, whereas appropriately constructing the priors reduces the bias (see also Table S1).

In order to consider the effect of model misspecification on parameter estimation, datasets are simulated under a model where parameters were drawn from distributions, yet priors are constructed assuming that these parameters have fixed values. Misspecification of the model in this way leads to an upward bias in the estimate of selection coefficients, and a downward bias in the estimated rate of selection (Figure 5). To account for this misspecification, the priors must be appropriately constructed, by allowing each locus within a given replicate dataset to also be drawn from distributions (see Methods). As shown in Figure 5, while the distribution of MAP estimates are more greatly dispersed when compared with Figure 4 (*e.g.*, under a fixed model the RMSE(*ŝ*) = 7.9E−06 for strong selection and large regions, and under a distributed model the RMSE(*ŝ*) = 1.11), the mean of the distribution nonetheless accurately reflects the means of *s*, 2*Nλ*, and *θ* (for the above two models, the RB(*ŝ*) are 0.12 and 0.57, respectively; Table S1). Additionally, for all estimated parameters, the relative bias is reduced for 50 kb relative to 500 bp regions.

For comparison, an alternate distributed parameter model was considered. As opposed to *s* being drawn from an exponential distribution for each locus, we model *s* being drawn from an exponential distribution for each selective event. Results between the two models are similar, though this case results in consistently smaller RMSEs (results under this alternative model, mirroring Figure 5, are given in Table S1). This result suggests that this alternative distribution model is intermediate between the two extreme cases examined here - fixed models and distributed locus-by-locus models. Despite the overall improvement gained by modeling distributed parameters in general, an important limitation is the assumption that the shape of the underlying distribution of each parameter is known.

The above simulations however, continue to assume a constant mutation rate among regions. In reality, the mutation rate may vary among loci, which may be a potential source of bias for the method [11]–[12]. Thus, in order to consider the possible effects of mutation rate variation, the distribution of variation at synonymous sites among loci in the Andolfatto (2007) dataset (see below) was taken as a proxy for mutation rate variation. We estimated the parameters for a Γ-distribution using the distribution of synonymous site divergence estimates across loci. Modeling this observed distribution with simulated data (*i.e.*, Γ(200,2.5); Figure S3), we found that the estimation was not affected and results resemble those of a fixed *θ* model (Figure S1, Figures 4–5). This result suggests that the variation in mutation rate observed in *D. melanogaster* is not widely dispersed enough to impact estimation, and is thus not likely to be biasing our estimated parameter values.

As there is relatively little variance at synonymous sites observed among regions in the Andolfatto (2007) dataset, data was simulated in which *θ* is much more widely dispersed (*i.e.*, Γ (10,50)), in order to determine the possible bias introduced by more extreme mutation rate variation. Importantly, under this model, estimation based upon 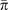 and SD(*π*) becomes strongly biased in the direction of estimating larger selection coefficients, as heterogeneity in mutation rate is artificially inflating the variance among loci (Figure S3). However, when estimation is based upon the means and SDs of *π*, *S*, *θ_H_*, and *ZnS*, results appear robust to mutation rate variation (for *π*-based estimation, the RB of *ŝ* = 8.95, for all statistics the RB = 0.51; Table S1). This is owing to the fact that while *π* is greatly impacted by this heterogeneity, other statistics, such as *ZnS*, have standard deviations that vary greatly between RHH models, yet are largely unaffected by mutation rate variation within any given model. Importantly, we only here consider regional variation in mutation rates and not site-to-site variation within genes (*e.g.*, CpG in mammals).

In summary, we propose that our estimator of recurrent hitchhiking model parameters that incorporates information from multiple summary statistics performs reasonably well. This method is preferable to a *π*-based approach both because it is more accurate and more robust to variation in mutation rate. The overall performance of the method will be greatly improved by the availability of genome-scale polymorphism data. An important point relevant to all of these models is that relatively simple adaptive models have been considered, and additional complexities such as recently increased or decreased rates of adaptation, variation in dominance of beneficial mutations, or selection from standing variation, have yet to be incorporated.

### An Application to Multi-Locus Data from *D. melanogaster*

Here we apply our approach to the multi-locus data set of Andolfatto (2007), who surveyed 137 X-linked regions from an East African population of *D. melanogaster* [11]. Though our performance evaluation of the method suggests that regions of this size are not ideal for estimation (the average region length in this dataset is 680 bps), they indicate at least the possibility of distinguishing weak from strong selection models, though such small regions cannot assure accurate parameter estimation. We estimated selection parameters both from 1) priors where these parameters are drawn from distributions (exp(*s*), exp(2*Nλ*) and N(*ρ*, *ρ*/2), and 2) in order to compare to previous estimation methods, priors that assume fixed values of *s*, *2Nλ* and *ρ*. The strength of selection for each sweep, *s*, is drawn from an exponential distribution (see Methods). We ignore variation in *θ* among loci as we have shown that this is not expected to significantly impact estimation (see above).

**Figure 6:**
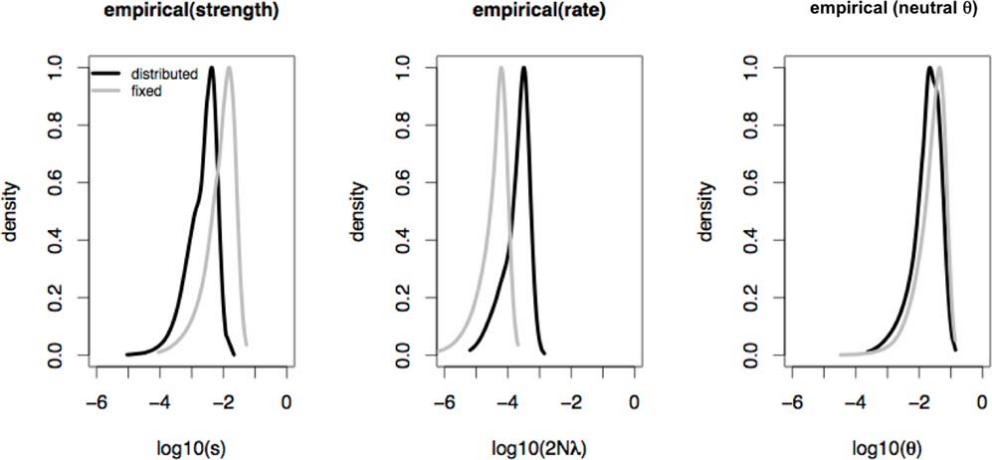
Marginal posterior distributions of *s*, 2*Nλ*, and *θ*, for the 137-locus dataset of [11], when estimation is based upon the means and SDs of *π*, *S*, *θ_H_* and *ZnS*. Results are given when the priors are constructed assuming fixed selection parameters, as well as when parameters for each locus are drawn from distributions (see Methods). In order to model the dataset under consideration, priors are constructed such that each replicate consists of 137 loci each of the observed length. *n* = 12, *ρ* = 0.121, and *N_e_* = 1.87^6^ (in accord with the estimates of [11]). Consistent with the simulation results, assuming a model in which selection coefficients are fixed leads to larger estimates of *ŝ*, and reduced estimates of 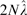.

Shown in Figure 6 are marginal posterior distributions for selection parameters (assuming distributed parameters, *ŝ* = 2E−03, 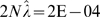, and 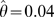 per site). Consistent with simulated data, parameter estimations assuming fixed values leads to considerably larger estimates of *ŝ*, and reduced estimates of 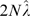 (Figure 6, *ŝ* = 0.01, 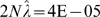, and 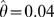 per site). It is thus important to emphasize that estimation will be sensitive to the underlying models chosen for the priors. Given that we expect these parameters to vary among loci, we consider the former estimate to perhaps be better, with the caveat that we lack precise knowledge of how these parameters are actually distributed (see Methods for more details). Interestingly, the large estimate of 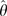 compared to previous studies [11]–[12] suggests a stronger mean reduction in genome variation due to hitchhiking (∼50%). Finally, it is additionally noteworthy that estimation does not necessarily need to be performed using the marginal posteriors as we have implemented here. For example, Figure S4 compares estimation between joint and marginal posteriors for our empirical dataset, and finds that while the estimates are similar, they are not identical. Understanding these differences, and better determining if estimation based upon joint posteriors may have any advantages, is a topic of future investigation.

### The Effect of Demography on the Estimator

An important consideration we have not addressed thus far is the impact of non-equilibrium demography, which may closely resemble sweep-like patterns of variation and may be expected to bias the estimator [*e.g.*, 27]–[28]. For instance, a strong population bottleneck exhibits many characteristics of a selection model – greatly increasing the variance of summary statistics, and specifically producing very negative values of the *H*-statistic [29]–[32]. In order to assess the potential bias induced by demography on the proposed estimator, we model two simple bottleneck models (BN1 and BN2) and a growth model (see Methods). BN1 and the growth model were fit to match the observed mean *π* and Tajima's *D*. BN2 was chosen specifically to match the observed CV(π). Under all three models, the posterior distributions are localized around weaker selection coefficients, and larger rates, than we estimate from the observed data, with estimation based upon distributed priors (MAP estimates given in Table 2).

**Table 2:** Comparing empirical estimates with estimated demographic models^a^.

	*ŝ* b	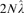 b
Empirical data	2E−3	2E−4
Growth^c^	7E−6 (6E−6 – 9E−6)	1E−2 (1E−2 – 2E−2)
BN1^c^	3E−5 (6E−6 – 7E−5)	7E−3 (1E−3 – 5E−2)
BN2^d^	5E−5 (7E−6 – 1E−4)	4E−3 (8E−4 – 1E−2)

a estimation is performed using distributed priors (exp(2*Nλ*) per locus, exp(*s*) per sweep – see Methods).

b MAP estimates (95% CI).

c model estimated to match the empirically observed *π* and Tajima's *D*.

d model estimated to match the empirically observed CV(*π*).

This result suggests both that, while the estimator is obviously sensitive to non-equilibrium demography, our empirical data is not easily explained by any of the demographic models considered (with the empirical estimates falling outside of the 95% CIs for the demographic models considered). This is particularly encouraging given that one of the bottleneck models, BN2, was chosen specifically to match the CV(π) that was observed for this dataset. Clearly, to minimize demographic effects, populations should be carefully chosen when possible. The dataset we have analyzed is from a putatively ancestral East African population that is believed to have been relatively demographically stable compared to non-African populations, which show signatures of a recent and severe bottleneck [18], [31]–[32]. Characterizing biases induced from a wider range of demographic models is a topic of future study, and will be important before performing estimation in other populations and species. One promising direction will likely take advantage of the observed correlation between *π_s_* and *K_a_* [11]–[12], which is difficult to explain under neutral demographic models. The incorporation of divergence data of this sort may increase the robustness of the estimator to non-equilibrium perturbations [12].

### Comparison with Existing Estimates of Recurrent Hitchhiking Parameters

Several other studies have attempted to estimate parameters under a recurrent hitchhiking model, and a discussion of how our estimates compare with those studies is of considerable interest. As previous studies assumed fixed values of *s*, 2*Nλ* and *ρ*, it is most appropriate to first compare these estimates with our “fixed value” estimation. Li and Stephan (2006) employed a sliding window likelihood ratio test using multi-locus polymorphism data and estimate that *ŝ*∼0.002 and 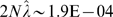 [18], which is similar to our estimates (Table 3). Their approach has a number of notable differences with ours: they co-estimate a growth model within their estimation procedure, use non-coding DNA rather than synonymous sites, and assume that all detectable sweeps have fixed immediately prior to sampling (*i.e.*, *τ* = 0). Given that our values of 2*Nλs *are quite similar, so is the expected level of reduction in genome variability (Table 3). Macpherson *et al.* (2007) used large-scale polymorphism data from six lines of *D. simulans* and estimate a strong average selection coefficient (*ŝ*∼0.01) [12], which is identical to our fixed value estimate. The bigger difference is in our estimates of 2*Nλ*, with our estimate being ∼4× larger. However, given that the dataset examined here is from *D. melanogaster*, there is no reason to necessarily anticipate that these estimates should match.

**Table 3:** Comparing estimates of recurrent hitchhiking model parameters in Drosophila.

	*ŝ*	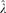	*Nˆ*	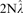	2Nλ*ŝ*
Li & Stephan 2006 [18]	0.002	1.1E−11	8.6E+06	1.9E−04	3.9E−07
Andolfatto 2007 [11]	1.2E−05	6.9E−10	1.9E+06	2.6E−03	2.6E−08
Macpherson *et al.* 2007 [12]	0.010	3.6E−12	1.5E+06	1.1E−05	1.1E−07
this study (fixed *s*,*λ*,*ρ*)	0.011	7.9E−12	2.5E+06	3.9E−05	4.3E−07
this study (distrib. *s*,*λ*,*ρ*)	0.002	4.2E−11	2.4E+06	2.0E−04	4.0E−07

It is noteworthy that our estimated selection coefficient is an order of magnitude smaller (and our estimate of the rate an order of magnitude larger) when we assume that *s*, 2*Nλ* and *ρ* are drawn from distributions rather than taking fixed values. Despite this, our estimated selection coefficient under the distributed model is still almost two orders of magnitude larger than Andolfatto's (2007) estimate [11]. Andolfatto's estimates of *s* and 2*Nλ* are particularly relevant, as we here examine the same dataset and arrive at quite different conclusions. The discord between estimates may arise from the fact that Andolfatto's estimate of *s* relies on estimating 2*Nλ* using the McDonald-Kreitman statistical framework [33]–[34]. However, we note that with short surveyed fragments, our estimator of *s* is somewhat upwardly biased (Figure 5) so it will be interesting to apply our method to larger genomic regions when that data becomes available.

Additionally, while Andolfatto (2007) and Macpherson *et al.* (2007) estimate a 20% average reduction in genome-wide variability, we estimate a considerably larger reduction (50%), which is more consistent with the estimate of 2*Nλs* of Li and Stephan (2006). This may to some extent explain Andolfatto's observation that the observed Tajima's *D* at synonymous sites is more negative than predicted by his estimates of *s* and 2*Nλ*. When we model a recurrent hitchhiking model with our estimated parameters, the average Tajima's *D* is −0.3, which is close to the observed average (−0.28). While a negative mean Tajima's *D* is usually interpreted in the context of demographic models (such as population growth, see for example [18]), it may instead imply that recurrent hitchhiking may be having a larger genome wide impact than previously appreciated.

### Conclusions

While common/weak and rare/strong recurrent positive selection result in similar average levels of genome variation on average (for 2*Nλs* = constant), rare/strong selection greatly increases the variance of common summary statistics relative to common weak selection. We demonstrate, using an ABC approach based upon this observation, that the rate and the strength of selection may accurately be estimated jointly. Though there is some power to differentiate parameters using existing data, our results strongly suggest that genome scale data will afford much better discriminatory power. Our study also highlights that learning more about how parameters such as *s*, 2*Nλ* and *ρ* are distributed among loci will be crucial for accurate parameter estimation.

## Methods

### Simulation of the Recurrent Hitchhiking Model

We use the recurrent selective sweep coalescent simulation machinery described in [24], with a modification to account for the stochastic trajectories of positively selected mutations in finite populations [11], [35]–[36]. Briefly, sweeps are occurring in the genome at a rate determined by 2*Nλ* = *Λ*, where λ is the rate of sweeps per generation [6],[8]. Following [24], selective sweeps are allowed both within the sampled region, as well as at linked sites. This distinction is significant, because for large simulated regions the probability of a sweep within the region may not be negligible for large *Λ*. The rate of sweeps within a region is thus M*Λ*, and as each sweep may affect up to *s/r_bp_* (from [6],[37]; which is equivalent to 4*Ns*/*ρ_bp_*), the rate considering both the sequenced and flanking regions becomes 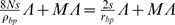, where *ρ_bp_* is the scaled recombination rate between base pairs and *M* is the size of the region in base pairs (see [6],[37] for details). With this, the expected waiting time between sweeps is 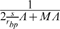 in 2*N* generations.

For the purposes of testing the proposed estimator, we evaluated models for *N_e_* = 10^6^, *θ* = 4*Nμ* = 0.01/site, and *ρ* = 4*Nr* = 0.2/site (*r* = 5E−08 per site per generation) and 0.1/site (*r* = 2.5E−08 per site per generation) in order to replicate Drosophila-like parameters ([32]; corresponding to values of *ρ/θ* = 20 and 10, respectively). The product *sλ* was set at 2.5E−13 in the case of *ρ/θ* = 10, and to 5E−13 for *ρ/θ* = 20. To replicate human-like parameters, we consider *N_e_* = 10^4^, *θ* = 0.002/site, and *ρ* = 0.002/site (*r* = 5E−08 per site per generation; corresponding to *ρ/θ* = 1) and *sλ* was set at 5E−11. In all cases, the sample size (*n*) = 25, and neutral variation is reduced to 60% of the neutral expectation. These calculations may be made from Eq.(5) of [7], which predicts the expected heterozygosity at linked neutral sites,
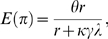
(1)where *θ* is the neutral population mutation rate, *r* is the unscaled recombination rate in Morgans per base pair per generation, *κ* is a constant ∼0.075, *γ* = 2*N_e_s* (where *s* is the selection coefficient), and *λ* is the rate of adaptive substitutions per site per generation. In most cases, simulated datasets consist of 10 50 kb regions or 1000 500 bp regions (which correspond to the same number of surveyed sites). 10,000 replicate datasets were generated under each model.

When simulating distributed rather than fixed values of *s*, 2*Nλ*, *θ*, and *ρ*, values for each region are drawn from a distribution (exp(*s*), exp(2*Nλ*), N(*ρ*, *ρ*/2) or exp(*ρ*). Thus, the value is fixed for an individual locus, but varies among loci. An alternative model was additionally examined, in which *s* is not fixed per locus, but rather is drawn from an exponential distribution for each selective event. These two separate models were chosen for two distinct purposes: 1) an exp(*s*) per locus is chosen for the performance simulations as it results in a large variance between loci. Thus, alongside the fixed parameter model, these comparisons represent two extremes; 2) an exp(*s*) per sweep is chosen when analyzing the empirical and demographic data, as we believe it better approximates biological reality (representing a model first introduced by Fisher). While the true underlying distributions are unknown, there is some biological data to draw from. For instance, observed *K_a_* among genes [11] is nearly exponentially distributed, implying that an exp(2*Nλ*) is a reasonable approximation. We model a normally distributed recombination rate for Drosophila-like parameters since heterogeneity in recombination rates is not believed to be large [38]. Additionally, recombination rate variation is minimized in the Andolfatto (2007) dataset analyzed here, as high recombination regions of the X were surveyed. For human-like parameters, we model an exponential recombination rate because recombination rate heterogeneity is more extreme [39]. When comparing between fixed and distributed models, a fixed value of *s* = 0.01 for example, is compared with a distributed model in which 0.01 is the mean of the exponential distribution from which the loci are drawn. In order to assess any bias which may be associated with variable mutation rates between regions, models were tested in which *θ*/locus is drawn from a Γ-distribution. Two Γ-distributions are examined, one matching the observed CV of synonymous site divergences among loci in the Andolfatto (2007) dataset analyzed here (Γ(200,2.5)), and one in which *θ* is very widely dispersed (Γ(10,50)).

In order to consider the performance of our method under non-equilibrium demographic models, we fit a simple bottleneck and growth model to the empirical data based on observed values of 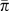 and the average Tajima's *D* (0.025/site and −0.28, respectively). Under both models, simulation parameters are thus scaled to mimic the observed values of these two statistics. As with above, *n* = 12, *ρ* = 0.1, *θ* = 0.01 and *N_e_* = 10^6^. Course grids under both models were simulated using the program *ms* [40]. We estimate a growth model in which growth rates were set to *α* = 50 at time *t* = 0.5 4*N* generations in the past, where *N(t)* = *N_0_*exp^−αt^. We estimate a bottleneck model that posits a stepwise reduction to 0.0001 of the population's former size beginning at *t_b_* = 0.5 and lasting 0.01 4*N* generations (BN1). In addition, a bottleneck model was selected to fit another feature of the data, the observed CV(*π*) (population reduced to 5.1% of its former size at time *t_b_* = 0.19 and lasting 0.01 4*N* generations; BN2). Estimation is performed using priors generated under a model in which parameters are distributed between loci (and *s* is distributed per sweep), as we argue that to be a more biologically relevant scenario compared to fixed parameter models.

### Parameter Estimation

To estimate the parameters *s*, 2*Nλ*, and *θ*, we relied upon their relationship with the means and standard deviations of common summary statistics. We take an approximate Bayesian (ABC) approach [41]–[44] to obtain marginal posterior distributions (estimation is also possible using joint posterior distributions, an example of this is discussed in the Results and given as a Supplement). Calculating our summary statistics (the means and SDs of *π*, *S*, *θ_H_* and *ZnS*) from the observed data, and from simulated data with parameters drawn from uniform priors, we implement the regression approach of [42]. Briefly, this involves fitting a local-linear regression of simulated parameter values to simulated summary statistics, and substituting the observed statistics into a regression equation. The prior distributions used were *s*∼Uniform (1.0E−06, 1.0), 2*Nλ*∼Uniform (1.0E−07, 1.0E−01), and *θ*∼Uniform (0.0001, 0.1), and the tolerance, δ = 0.001. Under a fixed selection parameter model, each draw from the prior represents the parameter value that is in common among all loci in a given dataset (*i.e.*, 1000 500 bp regions, or 10 50 kb regions). Under a distributed parameter model, each draw from the prior represents the mean of the distribution from which each locus in a given dataset will be drawn (or in the case of the alternative for modeling selection coefficients, a value of *s* is drawn for each sweep – see ‘simulation of the recurrent hitchhiking model’).

In order to determine the optimal combination of information, estimation was performed using all combinations of the mean and standard deviations of *π*, the number of segregating sites (*S*), *θ_H_*, Tajima's *D*, Fay and Wu's *H*, and *ZnS*. The combination of *π*, *S*, *θ_H_*, and *ZnS* was found to result in highly accurate and unbiased MAP estimates. Two statistics were utilized to evaluate the MAPs of *ŝ*, 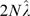 and 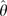. First, in order to measure any biases, the relative bias (RB) was determined from 1000 MAP estimates, as RB = Mean(*Xˆ*−*X*)/*X*. Second, in order to measure deviations from the expected values, the relative mean square error (RMSE) was determined as RMSE = Mean (*Xˆ*−*X*)^2^/*X* ^2^. The necessary code, and instructions for performing estimation, can be found at: http://www.molpopgen.org/.

### Empirical Data

We use the137 X-linked coding loci surveyed in [11]; Genbank accession numbers EU216760-EU218523. All loci were surveyed in 12 lines of *D. melanogaster* from a Zimbabwe population. For this analysis, only synonymous sites were considered. We summarized the mean average pairwise diversity, 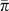, its standard deviation, SD(π), and the coefficient of variation, 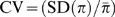, as well as the means and SDs of the number of segregating sites, *S*, *θ_H_* [25], Tajima's *D* [45], Fay and Wu's *H* [5], and *ZnS* [26], for synonymous sites across loci. Levels of synonymous polymorphism positively correlate with rates of divergence at synonymous sites [11]. To account for this, we also used partial regression corrected values of π at synonymous sites that account for variation in K_s_ [11]. We found that this had very little effect on 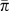 and SD(π) in this particular case.

## Supporting Information

Figure S1 Approximate Bayesian estimation of the strength and rate of selection as well as the neutral *θ*, when estimation is based upon the mean and SD of *π*. The model is one in which *s* and 2*Nλ* are fixed. For the strong selection case *s* = 1.0E−02 and 2*Nλ* = 2.0E−05, for weak selection *s* = 1.0E−04, and 2*Nλ* = 2.0E−03. *ρ* = 0.1/site and *θ* = 0.01/site. Shown are the distributions of 1000 MAP estimates. The dotted lines indicate the true values. The distributions for 10 50 kb region datasets are given in black, and for 1000 500 bp datasets in gray. As shown, the former affords more accurate estimation, and estimation is improved in general as *s* becomes large (see also Table S1).
(0.2 MB TIF)

Figure S2 The ratio CV to CV(equilibrium neutrality) for four values of *s*. The product *2Nλs* = 5E−07 for all panels. (A–D) Drosophila-like parameters: *ρ*/*θ* = 10 (*ρ* = 0.1/site, *θ* = 0.01/site), *ρ* = constant or Normal(0.1, 0.05). (E–H) Human-like parameters: *ρ*/*θ* = 1 (*ρ* = 0.002/site, *θ* = 0.002/site), *ρ* = constant or Exponential(0.1). (A,E) Exponential(*s*), Exponential(2*Nλ*), and *ρ* = Normal(0.1, 0.05). (B, F) Exponential(2*Nλ*), *s* = constant. (C, G) Exponential(*s*), 2*Nλ* = constant. (D, H) *ρ* = distributed, *s* = constant, 2*Nλ* = constant. The choice of exponentially distributed *ρ* for human-like parameters is motivated by evidence for greater heterogeneity in *ρ* relative to Drosophila [39]. Importantly, these models only represent one possible way of modeling distributions of *s* and 2*Nλ*, and alternative models may result in differing conclusions.
(0.2 MB TIF)

Figure S3 Approximate Bayesian estimation of the strength and rate of selection as well as the neutral *θ*, when estimation is based upon the means and SDs of *π*, *S*, *θ_H_* and *ZnS*, as well as with the mean and SD of *π* alone. The model is one in which *s* and 2*Nλ* are fixed, *s* = 1.0E−02, and 2*Nλ* = 2.0E−05, and *θ* is drawn from a Γ-distribution with mean 0.01 (given by dotted lines). *ρ* = 0.1. Shown are the distributions of 1000 MAP estimates. Results are given for *θ* drawn from two Γ-distributions, one meant to match the variance observed in the empirical dataset of Andolfatto (2007) (*i.e.*, Γ (200,2.5)), and the other simply for representing a very large variance (*i.e.*, Γ (10,50)). As shown, estimation based upon these multiple summary statistics appears to be robust to mutation rate variation, with *π*-based estimation being greatly biased (see also Table S1).
(0.2 MB TIF)

Figure S4 Joint posterior distributions of *s* and 2*Nλ*, for the 137-locus dataset of [11], when estimation is based upon the means and SDs of *π*, *S*, *θ_H_* and *ZnS*. Results are given when the priors are constructed assuming a distributed parameter model. In order to model the dataset under consideration, priors are constructed such that each replicate consists of 137 loci each of the observed length. *n* = 12, *ρ* = 0.121, and *N_e_* = 1.87^6^ (in accord with the estimates of [11]). The joint MAP is marked by the X, and the marginal MAPs (Figure 6) are given as dashed lines. As shown, estimation based upon joint posteriors is similar, though not identical, to the marginal posteriors.
(0.6 MB TIF)

Table S1 RMSE (RB).
(0.08 MB DOC)

## References

[1] Maynard Smith JM, Haigh J (1974). The hitchhiking effect of a favourable gene.. Genet Res.

[2] Harr B, Kauer M, Schlotterer C (2002). Hitchhiking mapping: a population-based fin-mapping strategy for adaptive mutation in Drosophila melanogaster.. Proc Natl Acad Sci USA.

[3] Stephan W, Wiehe THE, Lenz MW (1992). The effect of strongly selected substitutions on neutral polymorphisms: analytical results based on diffusion theory.. Theor Popul Biol.

[4] Simonsen KL, Churchill GA, Aquadro CF (1995). Properties of statistical tests of neutrality for DNA polymorphism data.. Genetics.

[5] Fay JC, Wu C-I (2000). Hitchhiking under positive Darwinian selection.. Genetics.

[6] Kaplan NL, Hudson RR, Langley CH (1989). The “hitchhiking effect” revisited.. Genetics.

[7] Wiehe THE, Stephan W (1993). Analysis of a genetic hitchhiking model, and its application to DNA polymorphism data from *Drosophila melanogaster*.. Mol Biol Evol.

[8] Braverman JM, Hudson RR, Kaplan NL, Langley CH, Stephan W (1995). The hitchhiking effect on the site frequency spectrum of DNA polymorphism.. Genetics.

[9] Przeworski M (2002). The signature of positive selection at randomly chosen loci.. Genetics.

[10] Begun DJ, Aquadro CF (1992). Levels of naturally occurring DNA polymorphism correlate with recombination rate in *D. melanogaster*.. Nature.

[11] Andolfatto P (2007). Hitchhiking effects of recurrent beneficial amino acid substitutions in the *Drosophila melanogaster* genome.. Genome Research.

[12] Macpherson JM, Sella G, Davis JC, Petrov DA (2007). Genomewide spatial correspondence between nonsynonymous divergence and neutral polymorphism reveals extensive adaptation in Drosophila.. Genetics.

[13] Kim Y (2006). Allele frequency distribution under recurrent selective sweeps.. Genetics.

[14] Sawyer SA, Hartl DL (1992). Population genetics of polymorphism and divergence.. Genetics.

[15] Smith NG, Eyre Walker A (2002). Adaptive protein evolution in Drosophila.. Nature.

[16] Eyre-Walker A (2006). The genomic rate of adaptive evolution.. Trends Ecol Evol.

[17] Sawyer SA, Parsch J, Zhang Z, Hartl DL (2007). Prevalence of positive selection among nearly neutral amino acid replacements in Drosophila.. Proc Natl Acad Sci USA.

[18] Li H, Stephan W (2006). Inferring the demographic history and rate of adaptive substitutions in Drosophila.. PLoS Genet.

[19] Andolfatto P (2005). Adaptive evolution of non-coding DNA in Drosophila.. Nature.

[20] Bachtrog D, Andolfatto P (2006). Selection, recombination and demographic history in *Drosophila miranda*.. Genetics.

[21] Ometto L, Glinka S, De Lorenzo D, Stephan W (2005). Inferring the effects of demography and selection on Drosophila melanogaster populations from a chromosome-wide scan of DNA variation.. Mol Biol Evol.

[22] Wright SI, Bi IV, Schroeder SG, Yamasaki M, Doebley JF (2005). The effects of artificial selection on the maize genome.. Science.

[23] Kim Y, Stephan W (2002). Detecting a local signature of genetic hitchhiking along a recombining chromosome.. Genetics.

[24] Jensen JD, Thornton K, Bustamante CD, Aquadro CF (2007). On the utility of linkage disequilibrium as a statistic for identifying targets of positive selection in non-equilibrium populations.. Genetics.

[25] Fu Y-X (1996). New statistical tests of neutrality for DNA samples from a population.. Genetics.

[26] Kelly JL (1997). A test on neutrality based on interlocus associations.. Genetics.

[27] Jensen JD, Kim Y, Bauer DuMont V, Aquadro CF, Bustamante CD (2005). Distinguishing between selective sweeps and demography using DNA polymorphism data.. Genetics.

[28] Thornton KR, Jensen JD, Becquet C, Andolfatto P (2007). Progress and prospects in mapping recent selection in the genome.. Heredity.

[29] McVean GA (2002). A genealogical interpretation of linkage disequilibrium.. Genetics.

[30] Lazzaro BP, Clark AG (2003). Molecular population genetics of inducible antibacterial peptide genes in *Drosophila melanoaster*.. Mol Biol Evol.

[31] Haddrill P, Thornton K, Andolfatto P, Charlesworth B (2005). Multilocus patterns of nucleotide variability and the demographc and selection history of *Drosophila melanogaster* populations.. Genome Res.

[32] Thornton KR, Andolfatto P (2006). Approximate Bayesian inference reveals evidence for a recent, severe bottleneck in a Netherlands population of Drosophila *melanogaster*.. Genetics.

[33] McDonald JH, Kreitman M (1991). Adaptive protein evolution at the Adh locus in Drosophila.. Nature.

[34] Bierne N, Eyre-Walker A (2004). The genomic rate of adaptive amino acid substitutions in Drosophila.. Mol Biol Evol.

[35] Coop G, Griffiths RC (2004). Ancestral inference on gene trees under selection.. Theor Pop Biol.

[36] Przeworski M, Coop G, Wall JD (2005). The signature of positive selection on standing genetics variation.. Evolution Int J Org Evolution.

[37] Durrett R, Schweinsberg J (2004). Approximating selective sweeps.. Theor Popul Biol.

[38] Cirulli ET, Kliman RM, Noor MA (2007). Fine-scale crossover rate heterogeneity in *Drosophila pseudoobscura*.. J Mol Evol.

[39] Coop G, Przeworski M (2007). An evolutionary view of human recombination.. Nat Rev Genet.

[40] Hudson RR (2002). Generating samples under a Wright-Fisher neutral model of genetic variation.. Bioinformatics.

[41] Pritchard JK, Seielstad MT, Perez-Lezaun A, Feldman MW (1999). Population growth of human Y chromosomes: a study of Y chromosome microsatellites.. Mol Biol Evol.

[42] Beaumont MA, Zhang W, Balding DJ (2002). Approximate Bayesian computation in population genetics.. Genetics.

[43] Marjoram P, Molitor J, Plagnol V, Tavare S (2003). Markov chain Monte Carlo without likelihoods.. Proc Natl Acad Sci USA.

[44] Przeworski M (2003). Estimating the time since the fixation of a beneficial allele.. Genetics.

[45] Tajima F (1989). Statistical methods for testing the neutral mutation hypothesis.. Genetics.

