# Supplementary figures and images for: An Approximate Bayesian Estimator Suggests Strong, Recurrent Selective Sweeps in Drosophila
Source: PLoS Genet. 2008 Sep 19;4(9):e1000198. doi: 10.1371/journal.pgen.1000198 (PMC2529407; doi:10.1371/journal.pgen.1000198)

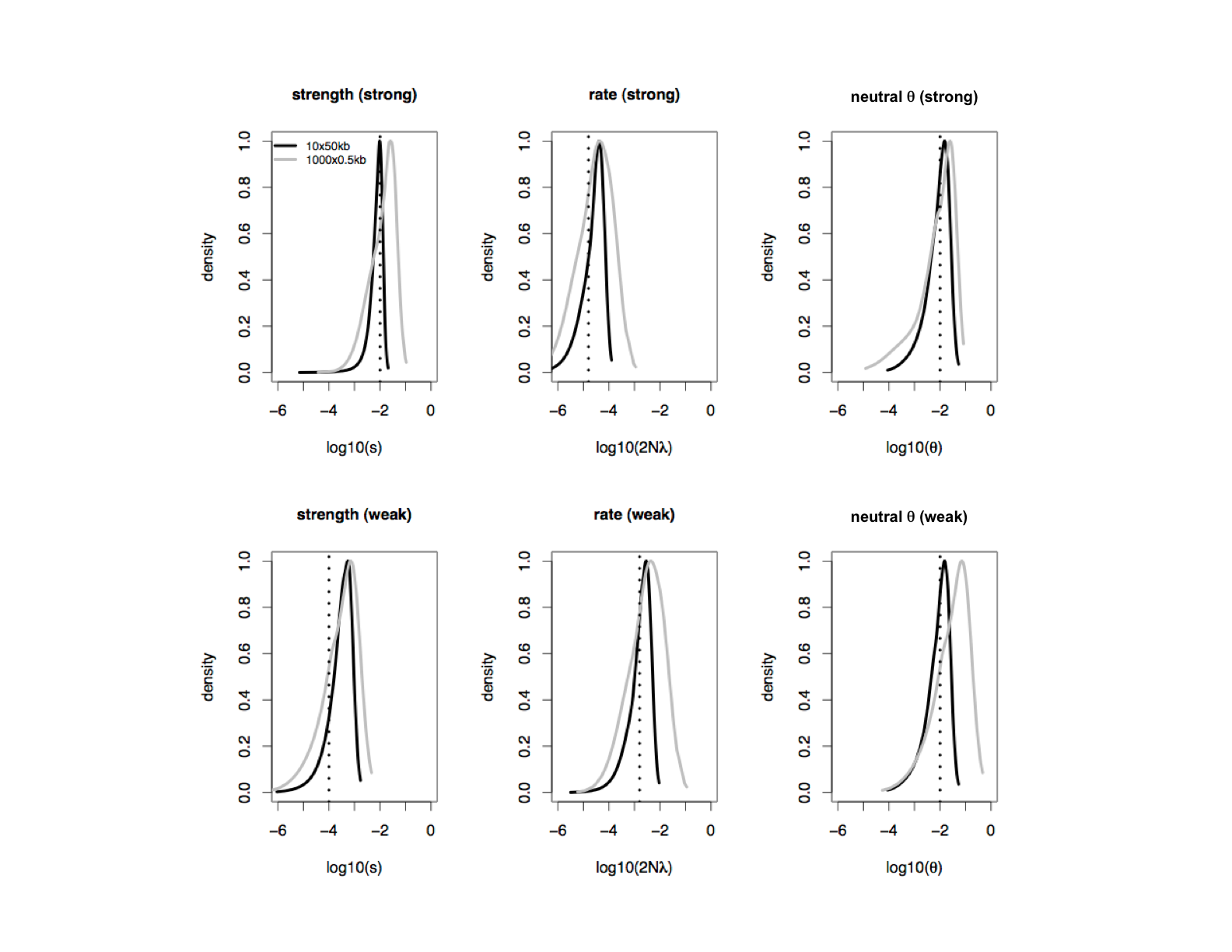

Supplement: Figure S1 — Approximate Bayesian estimation of the strength and rate of selection as well as the neutral θ, when estimation is based upon the mean and SD of π. The model is one in which s and 2Nλ are fixed. For the strong selection case s = 1.0E−02 and 2Nλ = 2.0E−05, for weak selection s = 1.0E−04, and 2Nλ = 2.0E−03. ρ = 0.1/site and θ = 0.01/site. Shown are the distributions of 1000 MAP estimates. The dotted lines indicate the true values. The distributions for 10 50 kb region datasets are given in black, and for 1000 500 bp datasets in gray. As shown, the former affords more accurate estimation, and estimation is improved in general as s becomes large (see also Table S1). (0.2 MB TIF) [file pgen.1000198.s001.tif]

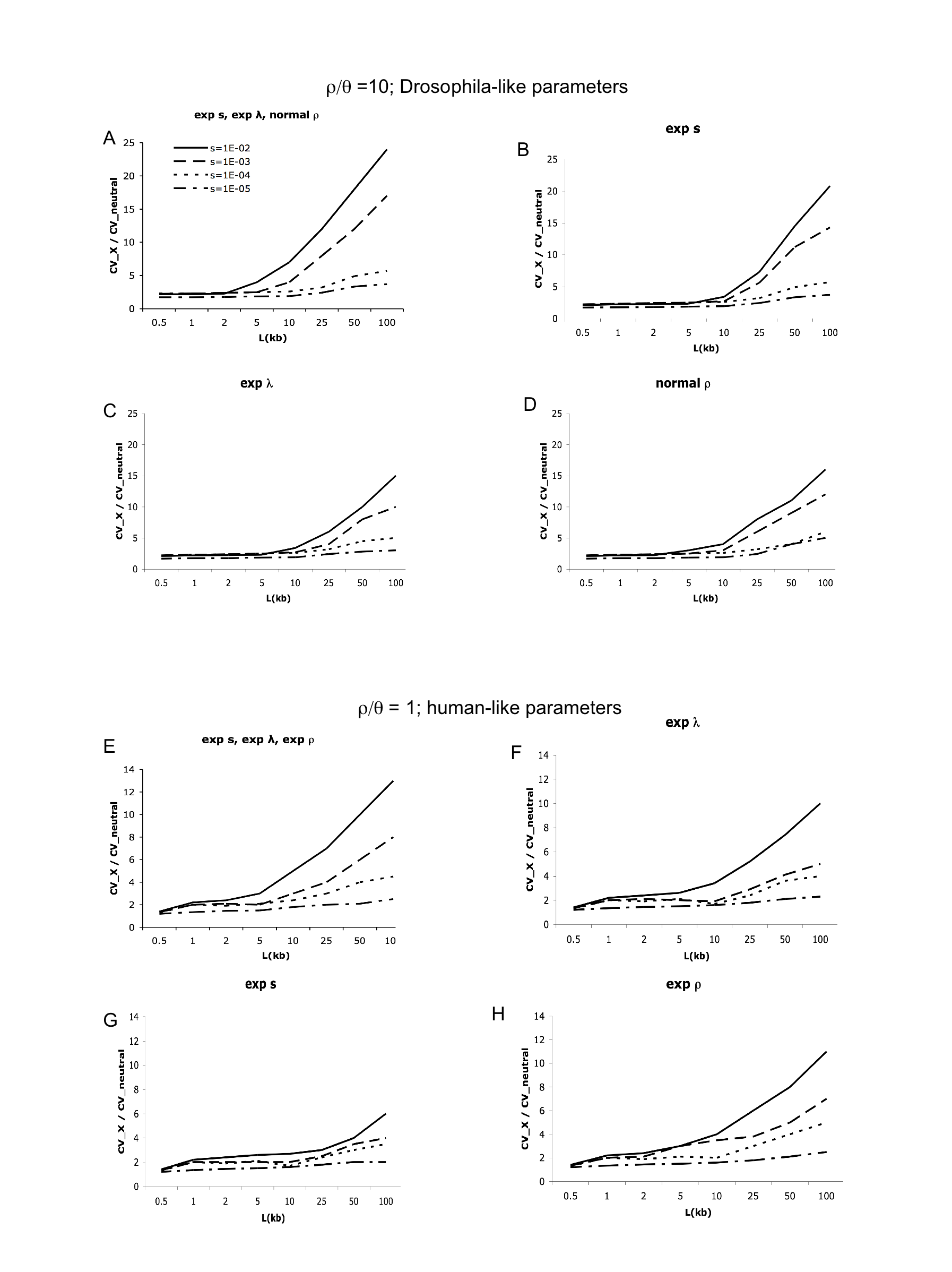

Supplement: Figure S2 — The ratio CV to CV(equilibrium neutrality) for four values of s. The product 2Nλs = 5E−07 for all panels. (A–D) Drosophila-like parameters: ρ/θ = 10 (ρ = 0.1/site, θ = 0.01/site), ρ = constant or Normal(0.1, 0.05). (E–H) Human-like parameters: ρ/θ = 1 (ρ = 0.002/site, θ = 0.002/site), ρ = constant or Exponential(0.1). (A,E) Exponential(s), Exponential(2Nλ), and ρ = Normal(0.1, 0.05). (B, F) Exponential(2Nλ), s = constant. (C, G) Exponential(s), 2Nλ = constant. (D, H) ρ = distributed, s = constant, 2Nλ = constant. The choice of exponentially distributed ρ for human-like parameters is motivated by evidence for greater heterogeneity in ρ relative to Drosophila [39]. Importantly, these models only represent one possible way of modeling distributions of s and 2Nλ, and alternative models may result in differing conclusions. (0.2 MB TIF) [file pgen.1000198.s002.tif]

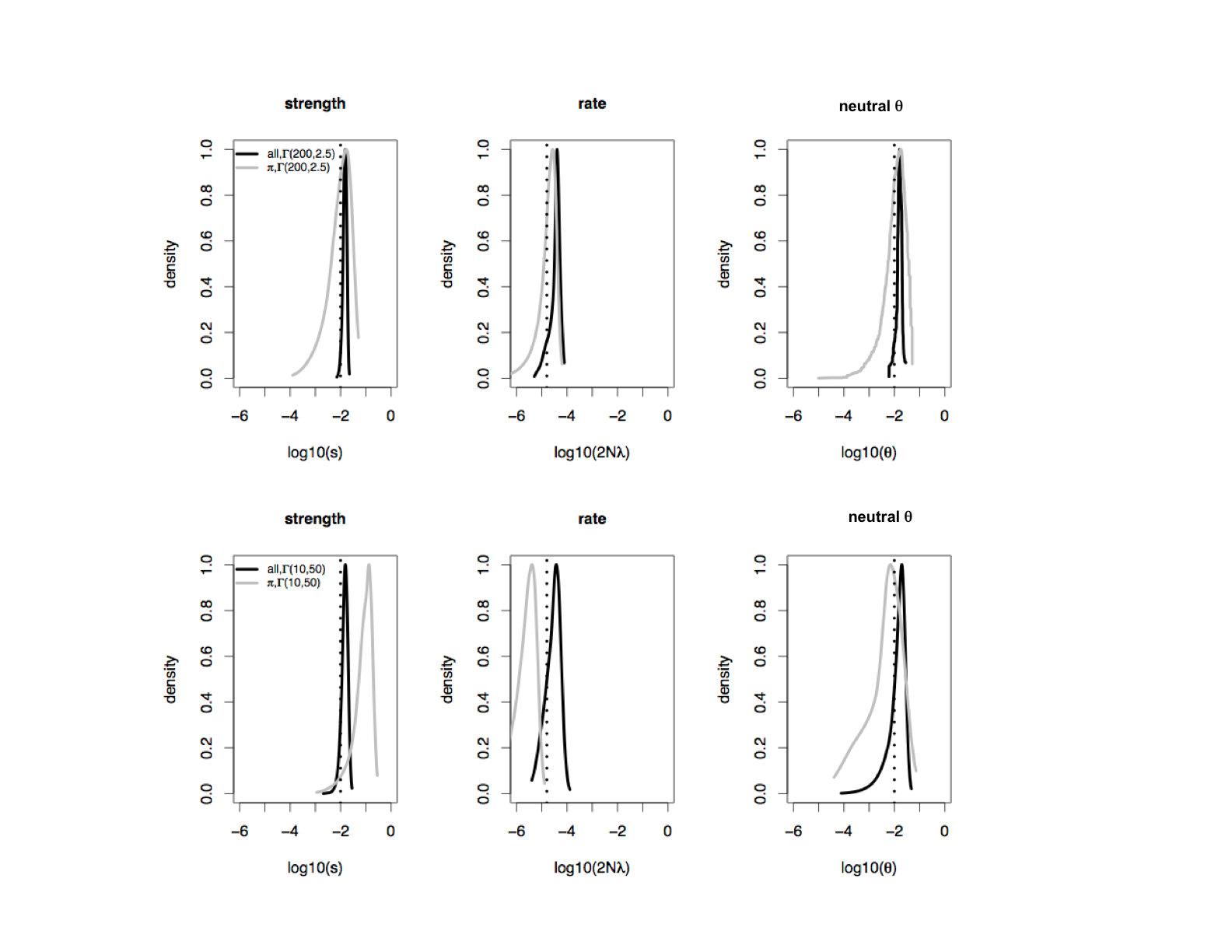

Supplement: Figure S3 — Approximate Bayesian estimation of the strength and rate of selection as well as the neutral θ, when estimation is based upon the means and SDs of π, S, θH and ZnS, as well as with the mean and SD of π alone. The model is one in which s and 2Nλ are fixed, s = 1.0E−02, and 2Nλ = 2.0E−05, and θ is drawn from a Γ-distribution with mean 0.01 (given by dotted lines). ρ = 0.1. Shown are the distributions of 1000 MAP estimates. Results are given for θ drawn from two Γ-distributions, one meant to match the variance observed in the empirical dataset of Andolfatto (2007) (i.e., Γ (200,2.5)), and the other simply for representing a very large variance (i.e., Γ (10,50)). As shown, estimation based upon these multiple summary statistics appears to be robust to mutation rate variation, with π-based estimation being greatly biased (see also Table S1). (0.2 MB TIF) [file pgen.1000198.s003.tif]

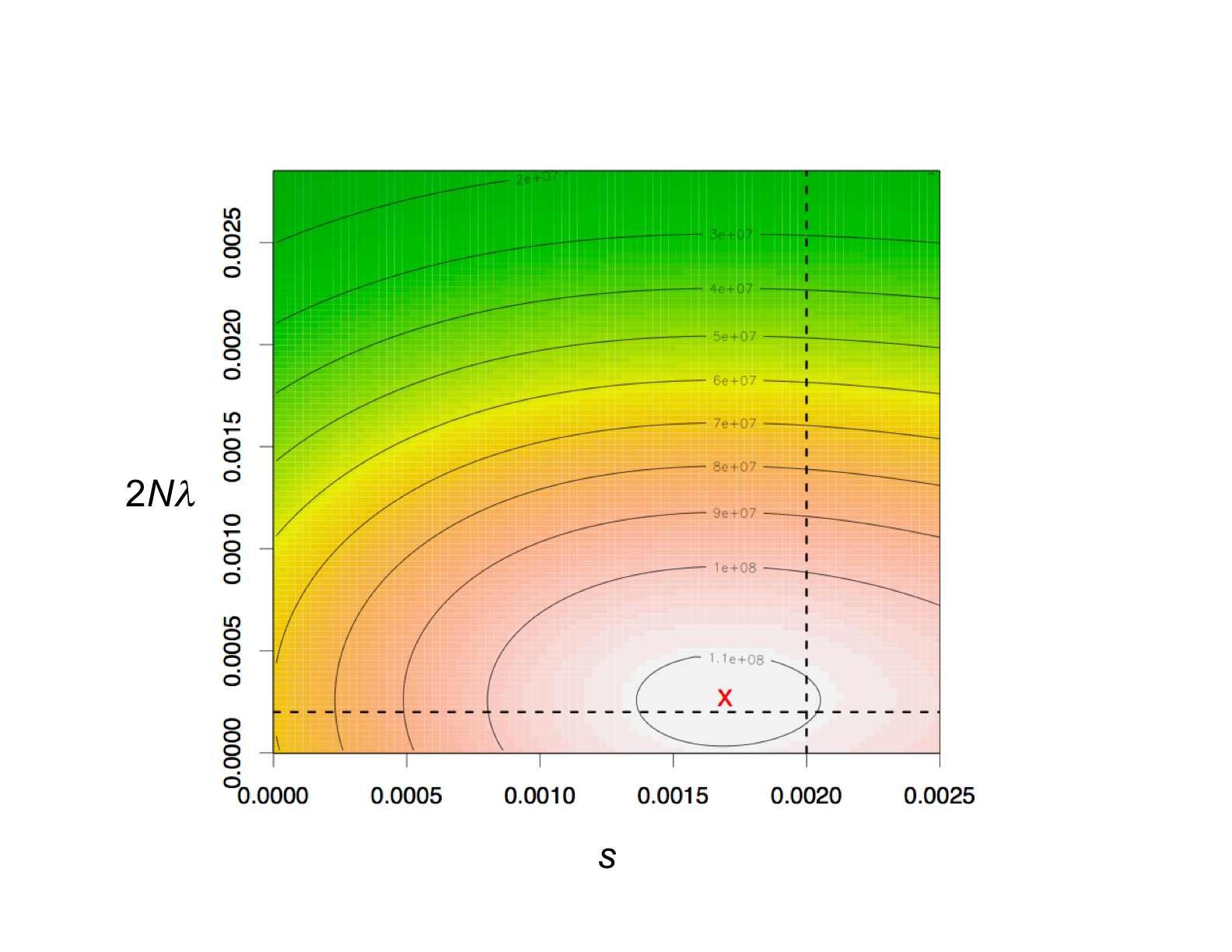

Supplement: Figure S4 — Joint posterior distributions of s and 2Nλ, for the 137-locus dataset of [11], when estimation is based upon the means and SDs of π, S, θH and ZnS. Results are given when the priors are constructed assuming a distributed parameter model. In order to model the dataset under consideration, priors are constructed such that each replicate consists of 137 loci each of the observed length. n = 12, ρ = 0.121, and Ne = 1.876 (in accord with the estimates of [11]). The joint MAP is marked by the X, and the marginal MAPs (Figure 6) are given as dashed lines. As shown, estimation based upon joint posteriors is similar, though not identical, to the marginal posteriors. (0.6 MB TIF) [file pgen.1000198.s004.tif]
