# Supplementary material for: An Approximate Bayesian Estimator Suggests Strong, Recurrent Selective Sweeps in Drosophila
Source: PLoS Genet. 2008 Sep 19;4(9):e1000198. doi: 10.1371/journal.pgen.1000198 (PMC2529407; doi:10.1371/journal.pgen.1000198)
Supplement: Table S1 — RMSE (RB). (0.08 MB DOC) [file pgen.1000198.s005.doc]

| Supplementary Table 1: RMSE (RB) | | | | |
| --- | --- | --- | --- | --- |
| source | model |  | 2N$\hat{\lambda}$ | θ |
| Supp Figure 1 | Strong 50kb | 0.18 (-0.28) | 0.75 (0.17) | 0.94 (0.22) |
|  | Weak 50kb | 20.9 (3.24) | 2.0 (0.69) | 0.92 (0.33) |
|  | Strong 500bp | 4.51 (1.01) | 3.3 (0.74) | 3.25 (0.75) |
|  | Weak 500bp | 111 (5.87) | 50.7 (2.9) | 123.0 (3.27) |
|  |  |  |  |  |
| Figure 4 | Strong 50kb | 7.9E-6 (0.12) | 4.4E-4 (0.052) | 0.52 (0.53) |
|  | Weak 50kb | 12.5 (2.66) | 0.66 (0.16) | 1.3 (0.34) |
|  | Strong 500bp | 2.81 (0.57) | 3.47 (0.50) | 4.52 (1.21) |
|  | Weak 500bp | 178 (6.24) | 5.21 (0.67) | 158 (7.42) |
|  |  |  |  |  |
| Figure 5 | Dist. 50kb | 1.11 (0.57) | 3.42 (1.04) | 0.17 (-0.18) |
|  | Fixed 50kb | 13.5 (2.54) | 0.59 (-0.73) | 4.5 (1.1) |
|  | Dist. 500bp | 2.75 (0.93) | 23.1 (2.65) | 16.7 (3.1) |
|  | Fixed 500bp | 222 (8.31) | 0.37 (-0.42) | 3.2 (0.81) |
|  |  |  |  |  |
| Alt. exp(s) model | Dist. 50kb | 0.88 (0.42) | 2.15 (0.88) | 0.43 (0.32) |
|  | Fixed 50kb | 12.6 (2.63) | 0.61 (0.23) | 2.2 (0.55) |
|  | Dist. 500bp | 1.63 (0.77) | 12.2 (1.77) | 8.16 (2.22) |
|  | Fixed 500bp | 199 (6.54) | 0.55 (0.39) | 5.6 (1.1) |
|  |  |  |  |  |
| Supp Figure 3 | all.Γ(200,2.5) | 0.31 (0.81) | 1.3 (0.83) | 0.43 (0.56) |
|  | π.Γ(200,2.5) | 1.64 (0.43) | 0.44 (0.073) | 1.65 (0.43) |
|  | all.Γ(10,50) | 0.43 (0.51) | 1.52 (0.65) | 0.54 (0.51) |
|  | π.Γ(10,50) | 118 (8.95) | 0.72 (-0.89) | 1.63 (0.0031) |
